# Dark-field chest X-ray imaging for the assessment of COVID-19-pneumonia

**DOI:** 10.1038/s43856-022-00215-3

**Published:** 2022-11-21

**Authors:** Manuela Frank, Florian T. Gassert, Theresa Urban, Konstantin Willer, Wolfgang Noichl, Rafael Schick, Manuel Schultheiss, Manuel Viermetz, Bernhard Gleich, Fabio De Marco, Julia Herzen, Thomas Koehler, Klaus Jürgen Engel, Bernhard Renger, Felix G. Gassert, Andreas Sauter, Alexander A. Fingerle, Bernhard Haller, Marcus R. Makowski, Daniela Pfeiffer, Franz Pfeiffer

**Affiliations:** 1 Chair of Biomedical Physics, Department of Physics, School of Natural Sciences, Technical University of Munich, 85748 Garching, Germany; 2 Munich Institute of Biomedical Engineering, Technical University of Munich, 85748 Garching, Germany; 3 Department of Diagnostic and Interventional Radiology, School of Medicine & Klinikum rechts der Isar, Technical University of Munich, 81675 München, Germany; 4 Philips Research, Hamburg, Germany; 5 Institute for Advanced Study, Technical University of Munich, 85748 Garching, Germany; 6 Institute of AI and Informatics in Medicine, School of Medicine & Klinikum rechts der Isar, Technical University of Munich, 81675 München, Germany

**Keywords:** Medical research, Diseases

## Abstract

**Background:**

Currently, alternative medical imaging methods for the assessment of pulmonary involvement in patients infected with COVID-19 are sought that combine a higher sensitivity than conventional (attenuation-based) chest radiography with a lower radiation dose than CT imaging.

**Methods:**

Sixty patients with COVID-19-associated lung changes in a CT scan and 40 subjects without pathologic lung changes visible in the CT scan were included (in total, 100, 59 male, mean age 58 ± 14 years). All patients gave written informed consent. We employed a clinical setup for grating-based dark-field chest radiography, obtaining both a dark-field and a conventional attenuation image in one image acquisition. Attenuation images alone, dark-field images alone, and both displayed simultaneously were assessed for the presence of COVID-19-associated lung changes on a scale from 1 to 6 (1 = surely not, 6 = surely) by four blinded radiologists. Statistical analysis was performed by evaluation of the area under the receiver–operator-characteristics curves (AUC) using Obuchowski’s method with a 0.05 level of significance.

**Results:**

We show that dark-field imaging has a higher sensitivity for COVID-19-pneumonia than attenuation-based imaging and that the combination of both is superior to one imaging modality alone. Furthermore, a quantitative image analysis shows a significant reduction of dark-field signals for COVID-19-patients.

**Conclusions:**

Dark-field imaging complements and improves conventional radiography for the visualisation and detection of COVID-19-pneumonia.

## Plain language summary

Computed tomography (CT) imaging uses X-rays to obtain images of the inside of the body. It is used to look at lung damage in patients with COVID-19. However, CT imaging exposes the patient to a considerable amount of radiation. As radiation exposure can lead to the development of cancer, exposure should be minimised. Conventional plain X-ray imaging uses lower amounts of radiation but lacks sensitivity. We used dark-field chest X-ray imaging, which also uses low amounts of radiation, to assess the lungs of patients with COVID-19. Radiologists identified pneumonia in patients more easily from dark-field images than from usual plain X-ray images. We anticipate dark-field X-ray imaging will be useful to follow-up patients suspected of having lung damage.

## Abstract (web summary)

Frank, Gassert et al. use dark-field chest X-ray imaging to assess COVID-19-pneumonia. Dark-field imaging has a higher sensitivity for COVID-19-pneumonia than attenuation-based imaging and provides an ultralow dose alternative to computed tomography imaging for that purpose.

## Introduction

The pandemic of severe acute respiratory syndrome coronavirus 2 (SARS-CoV-2) has led to a global medical, social, and economic crisis. The respective respiratory illness, coronavirus disease 2019 (COVID-19), has so far caused over six million deaths worldwide^1^.

At present, the reverse transcription-polymerase chain reaction (RT-PCR) test is the standard of reference for the definitive diagnosis of COVID-19^2,3^. While the Fleischner Society recommends computed tomography (CT) imaging in patients with COVID-19 and worsening respiratory status under certain conditions^4^, the use of CT as a primary screening tool is discouraged^5^, among other things, because it is associated with a rather high radiation dose. Therefore, alternative low-dose imaging techniques for the reliable evaluation and monitoring of pulmonary pathologies are highly desirable. This includes the potential application for follow-up assessment of patients suffering from long-COVID-syndrome, as radiation exposure reduction is crucial, especially in the setting of repetitive scans.

Dark-field X-ray imaging^6^ has been proposed as a new diagnostic tool for the assessment of micro-structural changes in lung parenchyma and has been positively evaluated for the imaging of various lung diseases in mouse models^7–9^ and first studies in humans^10,11^. In contrast to conventional X-ray imaging, which utilises the attenuation of X-rays in the specimen, X-ray dark-field contrast is generated by small-angle scattering of X-rays due to multiple refractions^6^. While the healthy lung consists of many refracting tissue–air-interfaces, generating a high dark-field signal, pulmonary disorders such as lung cancer, emphysema, fibrosis, or pneumonia decrease the dark-field signal by reducing the number of interfaces^7,8,12,13^. Previous works have already shown the feasibility of upscaling from the mouse model to human dimensions with a radiation dose comparable to conventional X-rays^10,11,14^. In this work, we describe several key advances of the dark-field chest X-ray imaging technique and its first application for the assessment of COVID-19-pneumonia in the human lung.

In a reader study, we found that dark-field imaging has a higher sensitivity for COVID-19-pneumonia than attenuation-based imaging and that the simultaneous display of both is superior to one imaging modality alone. Furthermore, a quantitative image analysis shows a significant reduction of dark-field signal for COVID-19-patients.

## Methods

### Hardware

The X-ray tube (MRC 200 0508, Royal Philips, The Netherlands) is operated at 70 kV in a pulsed mode at a frame rate of 30 Hz. The tube voltage was chosen because the most favourable combination of image quality, signal strength, and sharpness are present at 70 kV in both dark-field and attenuation images^15^. Tube current is adapted to each patient via his or her body mass index (BMI), as a high correlation between BMI and necessary tube current was found in a first-patient study. The flat-panel detector (Pixium FE 4343, Trixell, France) simultaneously acquires images at a time window of about 17 ms per frame. One scan consists of a maximum of 195 single frames taken in about 7 s. An additional mobile collimator (2 mm tungsten) is fixed to the source grating G0, limiting the actively irradiated area to the gratings, further reducing patient exposure. Downstream both collimators, an ionisation chamber (Diamentor CI, PTW, Germany) captures the entire radiation area to record the applied radiation dose of each scan. The effective patient dose is estimated from the measured dose area product^14^.

### Clinical image acquisition

As both the X-ray tube and detector are stationary, the patient is positioned within the beam path via a lifting platform inside the patient’s cabin. Both the positioning of the lifting platform and the movement of the interferometer can be operated with the control panel, located outside of the patient cabin. The shutters for vertical and horizontal collimation are also adjusted from there. The scan is triggered from the control room, and automatic breathing instructions are given. All images were acquired with full inspiration.

### Image reconstruction

Dark-field radiography with a three-grating Talbot–Lau interferometer works by analysing the fringe pattern produced by the phase grating G1 and sampled by the analyser grating G2. The source grating G0 is necessary to assure spatial coherence from a polychromatic X-ray source^16^. As the fringe pattern is too fine to be resolved by a conventional flat-panel detector, a slight mismatch in grating alignment is introduced to create moiré fringes on a resolvable scale for scanning image acquisition. By scanning the moiré fringes across the sample, a varying intensity is registered in every pixel for each image frame^17,18^. The so-called stepping curve can be extracted by a least-squares fit of a sinusoidal intensity model to the recorded intensities^6^. Additionally, scanning allows for the expansion of the field of view (FOV) in one direction, enabling the coverage of a human thorax. The difference between the patient scan and reference scan in the recorded intensities allows extraction of the attenuation and the dark-field image^19^. The high scanning speed introduces mechanical vibrations and, therefore, misalignment of the gratings, which we estimate with a maximum-likelihood method^20^. Still, the acquisition time is increased compared to conventional chest radiography, resulting in motion artefacts, especially around the heart contour and the aortic arch. By artificially narrowing the slot and reducing the number of frames for image extraction in affected areas, these motion artefacts can be strongly reduced^21^.

### Patient recruitment

#### Patients

Institutional Review Board (Ethics Commission of the Medical Faculty, Technical University of Munich, Germany; 587/16 S and 116/20 S) and national radiation protection agency approval Z5-22462/2-2017-021 and Z5–22464/2020-047-G) was obtained prior to this study. Patients gave their written informed consent prior to study participation.

#### COVID-19 patients

**Fig. 1:**
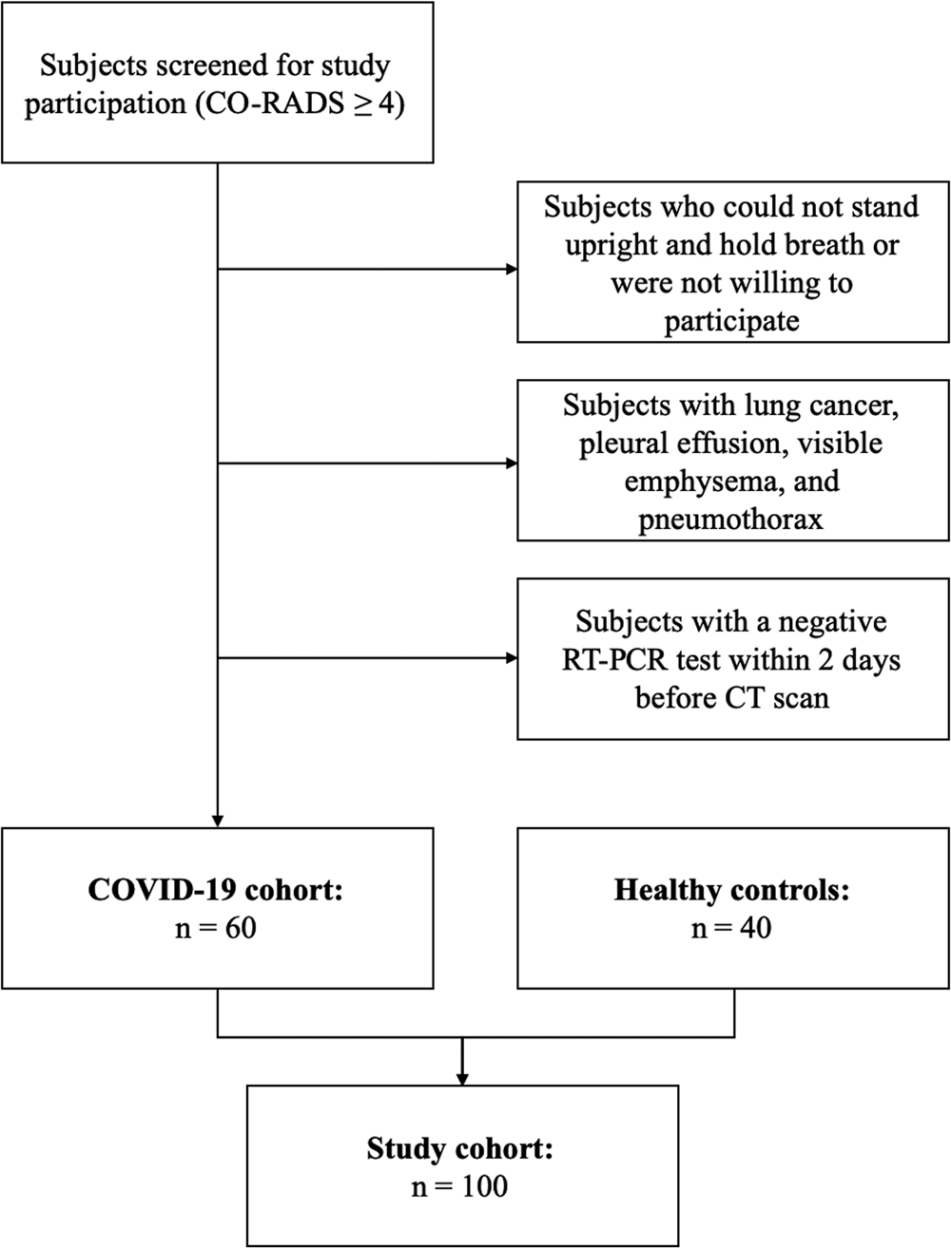
Flowchart illustrating patient selection. Subjects with a CO-RADS category ≥4 were screened for study participation. Taking into account the exclusion criteria, 60 participants were included in the COVID-19 cohort. Forty healthy subjects formed the control group.

Figure 1 illustrates COVID-19 patient selection. Between May 2020 and December 2020, patients of legal age (≥18 years) that underwent chest CT at our institution as part of their diagnostic workup and with a clinically suspected COVID-19 infection were screened for study participation. All CT images of potential study participants were analysed for COVID-19-pneumonia by two of three radiologists (F.T.G., A.S., A.A. with 2, 6 and 12 years of experience in chest CT imaging) immediately after the scan according to the CO-RADS assessment scheme for patients suspected of having COVID-19^22^.

Only patients with a CO-RADS category 4 (suspicious for COVID-19), 5 (typical for COVID-19), or 6 (RT-PCR positive for SARS-CoV-2, if patients had been tested before the CT scan) were included in this study. Other inclusion criteria were the ability to consent, to stand upright without help, and to hold breath for 7 s. Eligible patients were approached right after the CT scan.

Exclusion criteria were a negative RT-PCR test within 2 days before the CT scan, pregnancy, lung cancer, and pneumothorax. Sixty patients with suspected COVID-19 infection were included in this study.

#### Controls

Between October 2018 and January 2020, patients of legal age (≥18 years) that underwent chest CT at our institution as part of their diagnostic workup were screened for study participation. All CT images of potential study participants were analysed for pathologic lung changes by three radiologists (F.T.G., A.S., A.A.F. with 2, 6 and 12 years of experience in chest CT imaging). Inclusion criteria were a normal chest CT scan, the ability to consent, to stand upright without help, and to hold breath for 7 s. Eligible patients were approached right after the CT scan. Exclusion criteria were pregnancy, strong medical conditions, and changes in the lung tissue, such as cancer, pleural effusion, atelectasis, emphysema, infiltrates, ground glass opacities, and pneumothorax. Forty patients were included in the control group, previously reported by Gassert et al.^10^.

#### Computed tomography

CT was performed on one of three CT scanners (Philips iCT, Siemens SOMATOM, and Philips IQon Spectral CT) with the following parameters, according to routine clinical protocols: Reconstructed slice thickness, 0.625-0.9 mm; pixel spacing, 0.4/0.3 mm; pitch factor, 0.8/0.9; tube voltage (peak), 120 kV; modulated tube current, 125–350 mAs. Images were reformatted in 3 mm slice thickness using a lung-specific kernel.

### Image evaluation

Four radiologists (F.T.G., A.S., A.A.F., and D.P.) with different levels of experience in dark-field imaging (2, 5, 7, and 9 years) assessed only attenuation-based radiographs, only dark-field radiographs, and both displayed simultaneously for all patients. All readers were blinded to the group affiliation of images, and images were presented in random order. Readers used a PACS system and authorised monitors used in everyday clinical practice and were asked to rate the presence of COVID-19-pneumonia on a scale from 1 to 6 (1 = surely not, 2 = very unlikely, 3 = unlikely, 4 = likely, 5 = very likely, 6 = surely). Window settings were optimised for image illustration with the same window level for all images within each modality. Readers were allowed to adjust window levels at their convenience. Values 1–3 were counted as negatives, while values 4–6 were counted as positives.

Attenuation-based images were additionally evaluated by using the winning neural network of the SIIM-FISABIO-RSNA COVID-19 Detection Challenge^23^, which provides a probability for the presence of COVID-19-pneumonia for each patient.

The quantitative dark-field coefficient was calculated according to Gassert et al.^10^.

### Statistics and reproducibility

The area under the receiver operating characteristic (ROC) curve (AUC) was calculated for all three reading modalities, and AUC values were tested for differences with Obuchowski’s method for correlated and clustered ROC data^24^. Additionally, a z-test based on AUC values was used to determine whether the ratings of the two groups (healthy subjects and patients with COVID-19-pneumonia) differ within each reading modality. The averaged dark-field coefficients were tested for normal distribution using the Shapiro–Wilk-test, and only the coefficients of the healthy subjects were found to follow a normal distribution. Therefore, a two-sided Mann–Whitney-U-test was applied to determine whether the two groups (healthy subjects and patients with COVID-19-pneumonia) differ in average dark-field coefficient. The participant’s demographic parameters, age and weight were tested for significant differences between participants with COVID-19-pneumonia and the control group using Student’s *t*-test. For the parameter sex, a *χ*^2^ test was used. For all tests, a 0.05 level of significance was chosen. The inter-reader reliability for the presence of COVID-19-pneumonia was rated with Cohen’s weighted kappa (with quadratic weights).

As images were acquired in a clinical setting and COVID-19-associated lung changes change over time, reproduction of image acquisition is not feasible. Statistical analysis of acquired images, however, is reproducible.

### Reporting summary

Further information on research design is available in the Nature Portfolio Reporting Summary linked to this article.

## Results

### The Dark-field X-ray Prototype

**Fig. 2:**
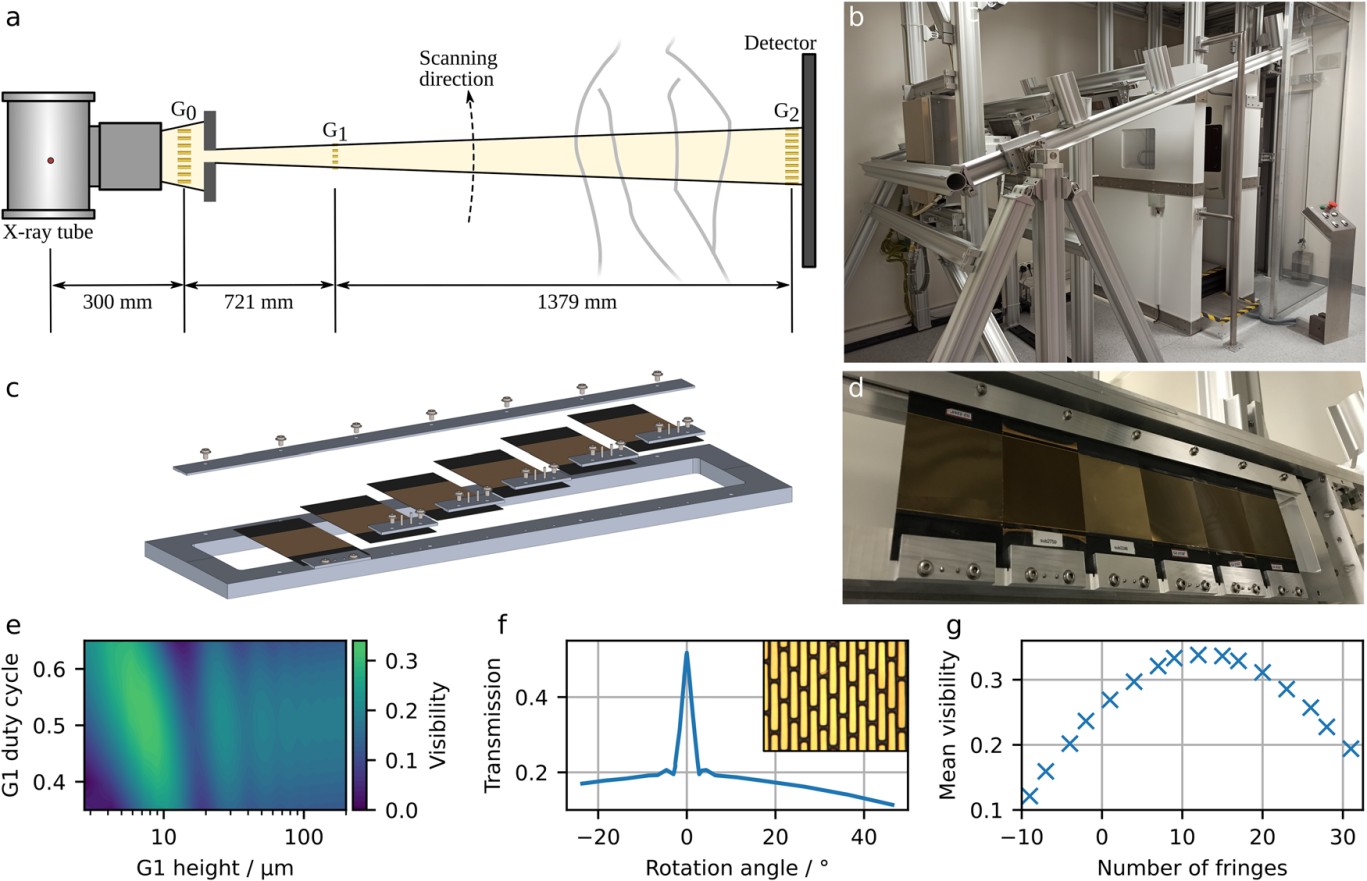
Design and implementation of the dark-field chest X-ray prototype. **a** Schematic and **b** photograph of the prototype system, combining a conventional radiography setup with a three-grating X-ray interferometer. **c** Schematic and **d** photograph of the G2 grating holder allowing individual positioning of the gratings as well as bending of the gratings according to the cone-beam geometry. Bending prevents the shadowing of the gold bars of the high aspect ratio gratings. **e** Simulated visibility for optimising of grating parameters such as duty cycle and height. The optimisation was carried out iteratively for all three gratings. **f** Angular X-ray transmission measurements and scanning electron microscopy image (inlet) for one of the grating structures. **g** Influence of a number of fringes per frame on mean interferometric visibility.

The first demonstrator system for clinical dark-field chest radiography in human patients consists of a conventional radiography system equipped with a three-grating X-ray interferometer^10,11^. A schematic of the system and a photograph are shown in Fig. 2a, b. The interferometer enables the simultaneous acquisition of both conventional attenuation and novel dark-field X-ray images. A medical X-ray generator is suspended from the ceiling to decouple the vibrations due to its anode rotations from the interferometer. The patient is standing upright within the interferometer inside a patient cabin to prevent injury and damage to the interferometer. A conventional flat-panel detector is mounted behind the patient cabin and the gratings, enabling a FOV of about 37 cm × 37 cm in the patient plane.

This large-scale system posed challenges previously not encountered. As the production of large area absorbing gratings with a high aspect ratio remains challenging, the analyser grating consists of six separate tiles. To prevent shadowing resulting from the cone beam geometry of the setup^25^, we designed a grating holder that allows bending the gratings along the axis of their lamellae via dowel pins, as well as individual positioning of the tiles. Figs. 2c and 2d depict a schematic and a photograph of the grating holder with its components, respectively.

Further, an in-depth optimisation of the gratings themselves is necessary for the optimal performance of the interferometer. Interferometric visibility is a measure of contrast in the interference pattern generated by the gratings. A higher initial visibility results in a better signal-to-noise (SNR) ratio in the final dark-field image^26^, which is retrieved from the visibility reduction induced by small-angle scattering. Depending on setup-specific parameters, such as the length of the interferometer and X-ray spectrum, the grating parameters, such as duty cycle and lamella height, need to be adapted for maximum visibility. Therefore, a propagation and simulation framework for X-ray grating interferometers was created to optimise these grating parameters^27^. An exemplary simulated visibility map for optimising G1 height and duty cycle can be found in Fig. 2d. In an iterative process, parameters of all three gratings were optimised regarding obtained visibility and producibility.

Ensuring the uniformity of the gratings’ duty cycle and lamella height is crucial for maximising the visibility of the setup and, thus, image quality. The newly developed angular X-ray transmission^28^ (Fig. 2f) allows for non-destructive and easily implementable parameter examination of gratings, and conventional SEM (Fig. 2f, inset) was used for local and surface-based analysis.

**Fig. 3:**
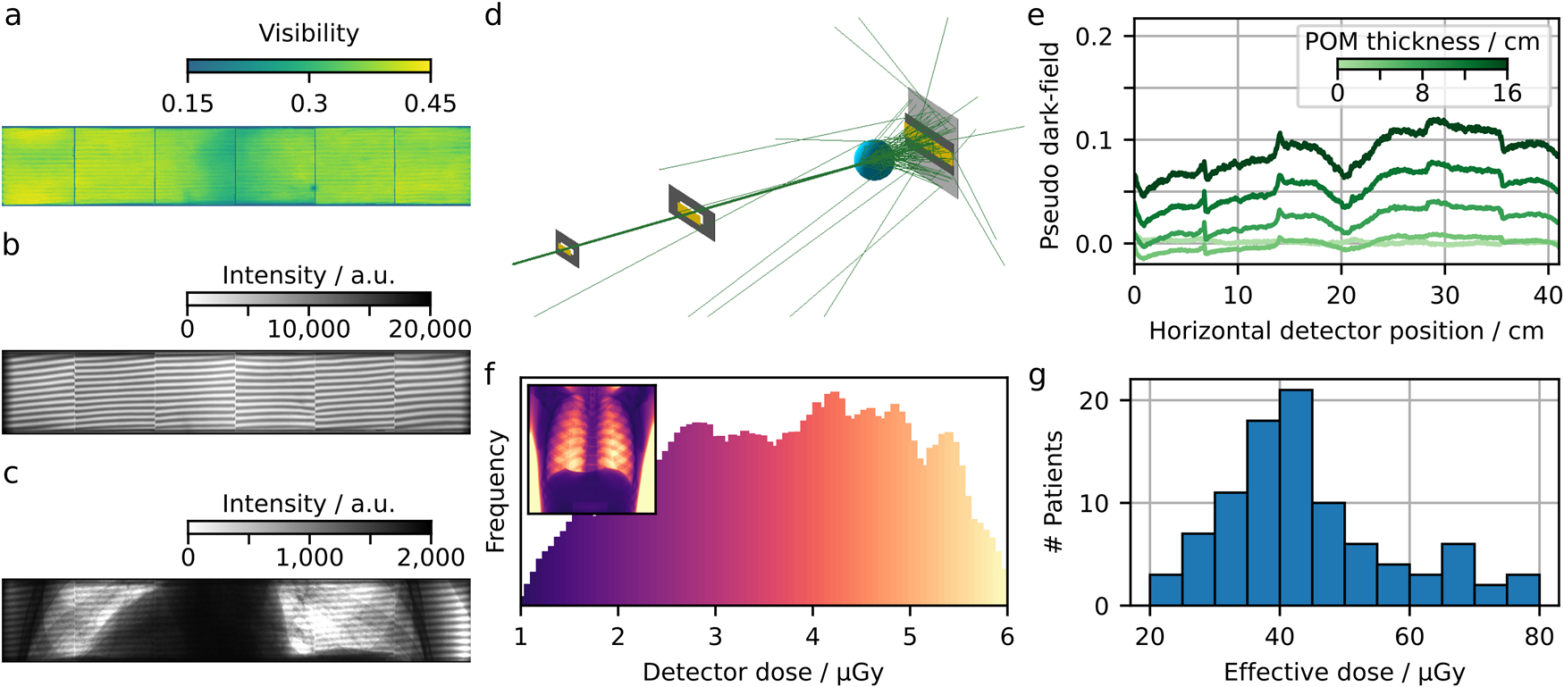
Optimising the interferometer for the detection of pulmonary pathologies. **a** Interferometric visibility map, which is a measure of the setup sensitivity. **b** One exemplary raw image frame without and **c** with a patient in the beam path. The changes in the interference pattern induced by a sample allow for (conventional) attenuation- and (novel) dark-field image extraction. **d** Monte-Carlo simulation of Compton scatter, adapted for setup-specific parameters. Based on such simulations, a correction for Compton scatter-induced dark-field contribution was applied. **e** Beam hardening induced by an equivalent material (here polyoxymethylene, POM). We implemented a beam hardening correction based on POM, assuming similar spectral behaviour between calibration material and patient. **f** Dose histogram of the lung region and detector dose map of a thorax phantom (inlet). **g** Histogram of effective dose deposited in the patients. For the patients described here, we report a mean (median) effective dose of 46.6 (41.7) µGy.

The interferometric visibility also depends on the number of moiré fringes, as depicted in Fig. 2g. The change in fringe number is achieved by adjusting the exact position of the phase grating within the beam path. A certain number of moiré fringes is observed because the grating periods do not exactly match the magnification condition of the cone beam geometry anymore. We found the highest visibility of 35% for 11 fringes and chose this configuration for clinical operation (see Fig. 3b).

### Optimising interferometer performance

Uniform visibility in the FOV ensures a constant dark-field sampling range, as the dark-field corresponds to a loss of visibility induced by a sample. The interferometric visibility without a sample in the beam path amounts to approximately 35%, as depicted for one interferometer position in Fig. 3a. The achieved visibility corresponds to the simulated visibility with the optimised grating parameters (cf. Fig. 2e). An exemplary raw data frame without a sample in the beam path can be seen in Fig. 3b. The moiré fringes, introduced by a small mismatch of the grating positions, are used to sample the intensity pattern with different relative grating positions when scanning the illuminated area over one pixel. Furthermore, the stitching gaps between the six grating tiles of the analyser grating subdivide the raw frame. By introducing a sample in the beam path, both the fringe pattern’s mean intensity as well as its contrast can change. Figure 3c depicts the raw data of a frame taken from an exemplary patient scan. Absorbing features appear dark, while the scattering lung reduces the contrast of the fringe pattern.

As the measured visibility in a polychromatic setup is a weighted average of photon-energy-dependent visibility, an attenuating object changes the measured visibility due to spectrally varying attenuation, resulting in beam hardening artefacts in dark-field images^29^. Figure 3e depicts horizontal profiles of the beam hardening-induced change in visibility and therefore generated pseudo-dark-field signal that occurs by introducing variant amounts of polyoxymethylene (POM) in the beam path. Due to inhomogeneities in the gratings as well as a variation of the source spectrum over the whole FOV, this effect depends on the spatial position on the detector. We see a strong dependence on the individual tiles of grating G2, with less pronounced changes in the scanning direction (not in the figure). We implemented a correction based on POM (aluminium), as its spectral absorption is similar to the one of soft tissue (bones). Using the approximation that the attenuation is caused by POM and aluminium in equal parts, we calculate the beam hardening-induced dark-field signal pixelwise, and subtract it from the measured dark-field signal.

Another effect distorting the visibility signal is Compton scatter, as it superimposes the recorded intensity pattern resulting in reduced contrast and therefore increased dark-field signal. A correction for Compton scatter-induced dark-field signal is implemented, based on the measured attenuation and Monte-Carlo simulations that are adapted from the Skyflow Software (Royal Philips, The Netherlands)^30,31^ for setup-specific parameters. From the attenuation image, the distribution of the material in the beam path approximated as water only is estimated. The intensity at the detector due to scattering is then calculated from the material distribution using scattering kernels, determined previously by Monte-Carlo simulations taking into account the spectrum and the setup geometry. An exemplary Compton scatters simulation is shown in Fig. 3d.

Within the approval process of the demonstrator system, clinical safety measures such as patient exposure and automatic shut-off to prevent overexposure were validated. An anthropomorphic thorax phantom was used for evaluating the detector dose in the examined lung (cf. Fig. 3f, Inlet depicts dose image of the whole FOV). The target detector dose was chosen such that the effective dose of the reference person amounts to 35 µSv for one scan in posterior-anterior orientation^14^.

The so obtained dose values (Fig. 3g) for examined patients range from about 20–80 μSv, depending mainly on the patient’s weight, with a mean value of 46.6 μSv and a median of 41.7 μSv, which is within reported chest radiography values^32^.

### COVID-19 in dark-field chest X-rays

#### Image appearance

A total of 100 patients (59 men, 41 women) were included, of which 40 were healthy controls, and 60 had COVID-19-pneumonia. The demographics of all study participants are listed in Table 1. No differences were found between healthy controls and patients with COVID-19-pneumonia regarding sex, age, and weight.

**Table 1:** Subject demographics.

Parameter	All	Healthy	COVID-19	*p*-value
Number of participants	100	40	60	
Men/women	59/41	25/15	34/26	0.56
Age in years	58 ± 14	61 ± 12	57 ± 15	0.18
Weight in kg	79 ± 16	79 ± 13	79 ± 16	0.89

Values are given as mean ± standard deviation. *P*-values for the significance of differences between the COVID-19 group and the healthy controls are listed in the very right column. The 40 healthy subjects were also included in Gassert et al.^10^ and Urban et al.^39^.

**Fig. 4:**
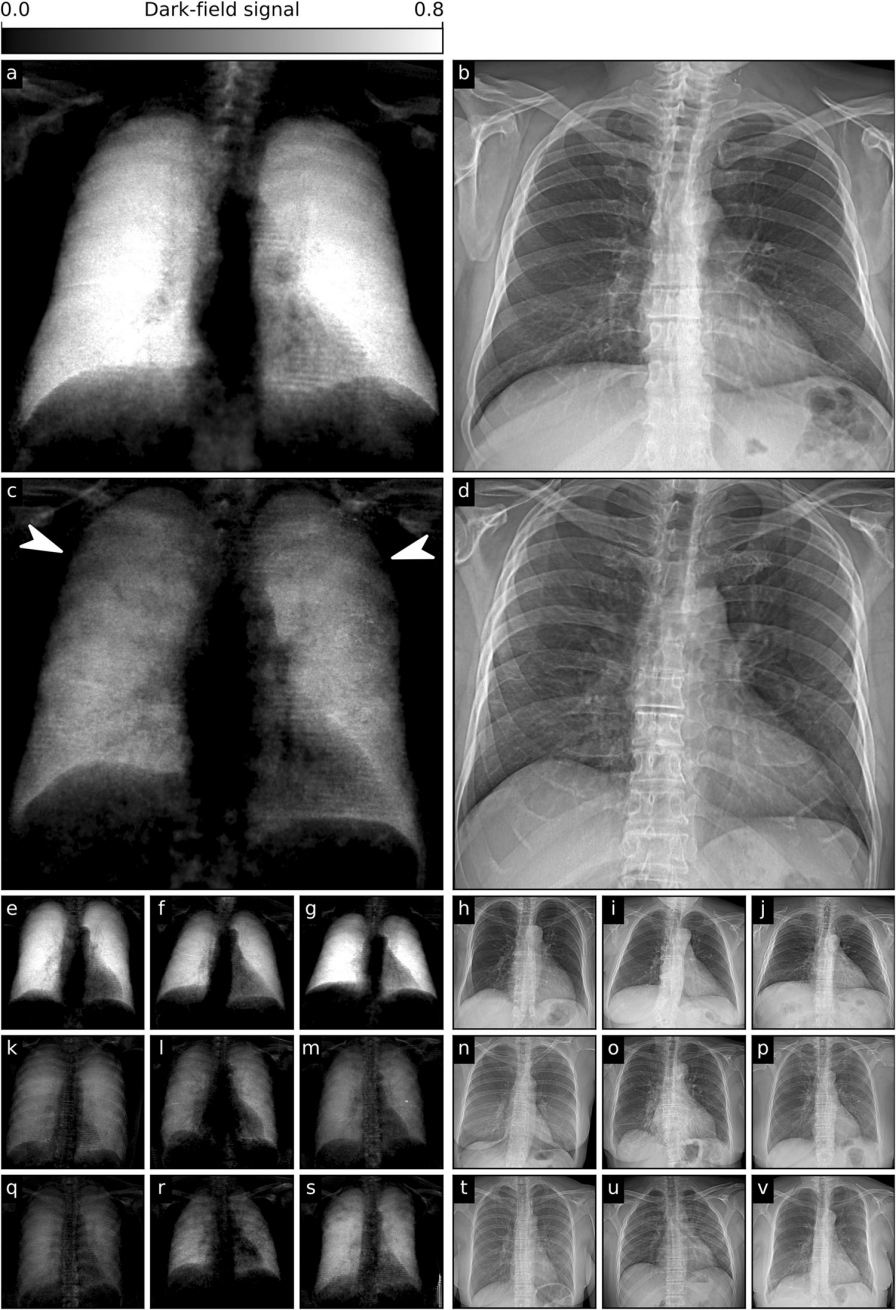
Dark-field and conventional chest X-rays of healthy and COVID-19-infected subjects. **a** Dark-field and **b** conventional (attenuation-based) chest radiographs of a healthy subject. The dark-field radiograph exhibits a strong, homogeneous dark-field signal. The respective attenuation-based image shows no apparent pathology. **c** Dark-field and **d** attenuation-based chest radiographs of a patient infected with COVID-19. Compared to the healthy subject, the infected patient shows an overall decrease of signal intensity. While the signal of the healthy subject is homogeneous, the dark-field signal of the infected patient appears inhomogeneous and patchy, especially in the periphery of the lung (arrowheads). **e**–**g** Additional exemplary dark-field radiographs of healthy subjects, **h**–**j**, respective attenuation-based radiographs. **k**–**v** More exemplary dark-field and respective attenuation-based images of patients infected with COVID-19. **k**–**m**, Dark-field images with a generally reduced signal intensity. **q-s**, Dark-field images with a rather inhomogeneous, patchy texture, predominantly in the periphery. **n**–**p** and **t**–**v** respective attenuation-based images in comparison, in which it is rather difficult to impossible to detect COVID-19-pneumonia.

Figure 4 shows the first X-ray dark-field imaging results on COVID-19 patients and healthy controls. Compared to dark-field images in healthy subjects, those in patients with typical COVID-19-pneumonia in the CT scan showed an overall decrease of the dark-field signal. While dark-field images in healthy subjects exhibit a rather homogeneous structure^10^, images of COVID-19 patients appear rather inhomogeneous and patchy, especially in the lung periphery, corresponding well to ground glass opacities in the respective CT scan (cf. Supplementary Fig. 1). While changes are obvious in dark-field images, conventional X-ray images of healthy subjects and infected patients are difficult to distinguish.

#### Reader study and quantitative analysis

**Fig. 5:**
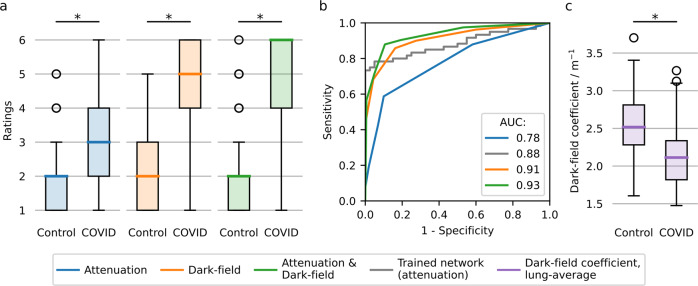
Results of clinical evaluation and statistical analysis. **a** Box plot of reader scores for both healthy subjects (*n* = 40) and infected patients (*n* = 60) in dark-field-based, attenuation-based, and dark-field & attenuation-based readings. **b** Receiver operator characteristic (ROC) analysis for the respective modalities for the differentiation between infected patients (*n* = 60) and healthy subjects (*n* = 40). Additionally, an AI algorithm (convolutional neural network) for COVID-19-pneumonia detection was applied for attenuation image evaluation. Area under the curve (AUC) values were 0.78 (attenuation), 0.88 (trained network on attenuation), 0.91 (dark-field), and 0.93 (dark-field & attenuation), respectively. **c** Objective, quantitative image analysis, showing the average dark-field coefficient (integrated over the whole lung area and evaluated after segmentation) for the lungs of healthy subjects (*n* = 40) and infected patients (*n* = 52) in a box plot. Significant differences are indicated by asterisks: *, p < 0.05. Abbreviations: *AUC*, area under the curve. Boxes in the box plots extend from the lower to upper quartile values of the data, with a line at the median. The whiskers extend to 1.5 times the interquartile range (Q3–Q1). Small circles represent flier points past the end of the whiskers. Underlying data can be found in Supplementary Data 1.

To evaluate the potential clinical impact, we performed a reader study for the detection of COVID-19-pneumonia on both attenuation-based and dark-field images alone, as well as both images, displayed simultaneously. The ratings for the presence of COVID-19-pneumonia in healthy subjects and patients with COVID-19-pneumonia in the CT scan did show a highly significant difference for all displayed variations, attenuation-based, dark-field-based imaging, and the combination of both (*p* < 0.05 for all) (Fig. 5a). Overall rating values for the presence of COVID-19-pneumonia in infected patients were substantially higher for dark-field imaging (4.84 ± 1.39) compared to attenuation-based imaging (3.16 ± 1.46). Additionally, rating values for infected patients were higher for the combination of dark-field-based and (conventional) attenuation-based imaging (5.04 ± 1.37) compared to dark-field-based imaging alone. In a ROC analysis for the differentiation between infected patients and healthy subjects, the effect size expressed as the area under the ROC curve (AUC) was 0.78 (95% confidence interval (CI): 0.73–0.83) for attenuation-based radiographs, 0.91 (95% CI: 0.88–0.94) for dark-field images and 0.93 (95% CI: 0.91–0.96) for the combination of both (Fig. 5b). By including dark-field images, AUC values were significantly higher compared to attenuation images only (*p* = 3.9e−6 for dark-field alone, *p* = 3.5e−9 for combination).

For comparison, we additionally applied the winning neural network of the SIIM-FISABIO-RSNA COVID-19 Detection Challenge^23^, trained on conventional attenuation images, to the attenuation images of both the COVID-19 patients and healthy controls. In this setting, an AUC value of 0.88 was achieved, which can also be found in the literature^33^. This value was higher than the AUC achieved by readers on the same images. However, compared to the trained network, readers achieved an even higher AUC when reading dark-field images alone or the combination of both imaging modalities (Fig. 5b).

The overall sensitivity for COVID-19-pneumonia was 0.43 (95% CI: 0.38–0.48) for attenuation-based images, 0.86 (95% CI: 0.80–0.92) for dark-field images and 0.88 (95% CI: 0.82–0.94) for the combination of both. Respective specificities and accuracies, also on an individual reader basis, are shown in Supplementary Table 1. The inter-rater reliability between the readers ranged from 0.16 – 0.42 for attenuation-based imaging, 0.56–0.67 for dark-field imaging and 0.48–0.74 for the combination of both. Reader-specific reliability scores are provided in Supplementary Table 2. The average rating of image quality over all readers was 4.97 ± 0.99 for dark-field and 5.35 ± 0.66 for attenuation-based imaging.

For a quantitative analysis of the dark-field signal, we calculated the average dark-field coefficient of every patient’s lung, corresponding to the average dark-field signal generated per path length through the lung parenchyma^10^. In the case of a collimation that covered part of the lungs, the lung volume and hence the dark-field coefficient, could not be calculated, resulting in a reduced number of participants in the COVID-19 cohort (*n* = 52). The average dark-field coefficient was significantly lower in patients infected with COVID-19 (2.15 ± 0.44 m^−1^) compared to healthy subjects (2.53 ± 0.44 m^−^^1^, *p* = 8.6e−5), as depicted in Fig. 5c.

The scores of the performed reader study, as well as the dark-field coefficients, are listed in Supplementary Data 1.

## Discussion

In this study, we present the first application of the recently developed dark-field X-ray imaging technology for the assessment of COVID-19-pneumonia and demonstrate its superiority over conventional radiography. This essentially introduces a low-radiation, medical imaging alternative to present CT imaging for COVID-19-pneumonia detection and therapy follow-up. Along with detailed subjective and objective clinical evaluation results, we also present some of the key technological improvements, which were necessary to optimise the system for imaging of pulmonary pathologies such as COVID-19-associated lung changes.

While we have achieved promising first results, some limitations of our approach and study exist. On the technological side, it first has to be noted that due to the restricted available grating sizes, the setup was realised as a slot scanning system. While this compromise allowed the first realisation of a clinical dark-field radiography study on COVID-19-pneumonia, future hardware improvements, such as the fabrication of full-field gratings, could enhance the current realisation by eliminating the scanning procedure and thus simplifying the setup. Gratings with larger aspect ratios could increase interferometric visibility, leading to a higher SNR ratio in dark-field images or lower effective doses. Also, gratings with smaller periods would increase the sensitivity of the setup, enabling the use of higher tube voltages and thus further decreasing the effective patient dose.

From a more clinical perspective, we showed that X-ray dark-field chest imaging is a fast, low-dose technique that yields both a conventional attenuation image and a novel, complementary dark-field image. It allows for the reliable detection of COVID-19-pneumonia and is, in that respect, superior to conventional radiography. For the latter, our results are in line with a previous study by Self et al.^34^, who found a similar sensitivity for the detection of pulmonary opacities in conventional attenuation radiographs as we found for attenuation images alone. In the performed reader study, the simultaneous presentation of both attenuation images and dark-field images yielded an even higher sensitivity compared to each imaging technique individually. The combined information from both attenuation and dark-field images provide an even better picture of the ventilation situation of the lung, also reflected by the higher inter-rater reliability for the combination of both imaging modalities compared to each imaging modality separately. Even though the achieved sensitivity when reading both modalities is not as high as in CT imaging^34^, it is still reasonably high and comes with only a fraction of the dose. We included only patients with moderate courses of the disease that were able to stand upright and hold their breath for the duration of the scan. These patients could be clearly distinguished from healthy controls, underlining the potential of the technique to detect even minor lung changes such as ground glass opacities.

While the evaluation of attenuation images was enhanced by a trained neural network, the reader-based assessment of dark-field images outperforms the algorithm. Finally, we are confident that there is potential to further enhance these results by applying artificial intelligence on dark-field images once a sufficiently large number of cases is available for training.

The patient study also exhibits some limitations. The study cohort, comprising 100 subjects, is presently relatively small, and the technique must be further evaluated in future studies with larger cohorts. In this context, another drawback is also that potential pulmonary comorbidities in COVID-19 patients are not taken into account, while the control group comprised only healthy subjects without any pulmonary disorders. While this initial pilot study aimed at evaluating the accuracy of X-ray dark-field imaging for the detection of COVID-19-pneumonia compared to pulmonary healthy subjects, future studies must be performed to evaluate the technique for the assessment of the lung when other pathologies are present.

Moreover, as the average dark-field coefficient is a measure of the alveolar integrity, it decreases with the presence of COVID-19-pneumonia. Currently, no pixel-based analysis is available, as the projected lung thickness in each pixel, which is necessary for the normalisation of the dark-field signal with the respective lung thickness, remains unknown. Therefore, only the average dark-field coefficient of the whole lung is available as the total signal is normalised with the lung volume. This leads to only a small reduction of the average dark-field coefficient in the presence of beginning and localised pneumonia, while the dark-field images show a distinct localised signal decrease in these cases. Whereas the quantitative analysis does not allow for the assessment of local changes in dark-field signals, radiologists may detect patterns of local signal losses. Future studies are needed to analyse the dark-field signal locally and therefore allow for a quantitative assessment of the alveolar integrity on a pixel basis.

While further technological improvements might enable a more accurate assessment of pulmonary pathologies in the future, more clinical studies are needed to evaluate the technique’s potential for lung imaging. Dark-field imaging might, for example, also be suitable for disease and treatment monitoring of COVID-19 patients due to the obtained image-based information on the lung’s alveolar condition at a low effective patient dose. With constantly new variants increasingly leading to higher infection rates^35^ and severe courses in younger patients^36^, dark-field imaging might be a low-radiation alternative for disease monitoring, especially in patients where repetitive CT scans should be avoided. Low-dose imaging techniques such as dark-field radiography are also highly desirable for the assessment of pulmonary involvement in patients with long-COVID-syndrome. However, this potential use case of the presented technique is yet to be evaluated. Nevertheless, the presented study highlights the potential of dark-field chest X-ray imaging for the assessment of COVID-19-pneumonia and shows that it might therefore be a promising new tool in the fight against the SARS-CoV-2 pandemic.

## Supplementary information

Description of Additional Supplementary Files

Supplementary Information

Supplementary Data 1

Reporting Summary

## Data Availability

The underlying data of Fig. 5 can be found in Supplementary Data 1. Other underlying data used in the evaluation of this study can be provided without patient identification upon reasonable request to researchers affiliated with accredited research institutions after entering a signed data access agreement. Proposals are required to address scientific questions and will be reviewed individually. Please direct your request to manuela.frank@tum.de.

## Code availability

Statistical analysis was performed with Python (version 3.8.5), specifically using the packages NumPy (version 1.20.2)^37^ and SciPy (version 1.5.4)^38^, as well as R (version 4.1.1) for the ROC analysis as described in the Methods section. We used the code available at https://www.kaggle.com/code/nguyenbadung/siim-covid19-2021/notebook?scriptVersionId=69474844.
