# Supplementary material for: Dark-field chest X-ray imaging for the assessment of COVID-19-pneumonia
Source: Commun Med (Lond). 2022 Nov 21;2:147. doi: 10.1038/s43856-022-00215-3 (PMC9678896; doi:10.1038/s43856-022-00215-3)
Supplement: Supplementary file 1 — Description of Additional Supplementary Files [file 43856_2022_215_MOESM1_ESM.pdf]

Description of Additional Supplementary Files

File Name: Supplementary Data 1

Description: Data used for statistical evaluation
