# Supplementary material for: Dark-field chest X-ray imaging for the assessment of COVID-19-pneumonia
Source: Commun Med (Lond). 2022 Nov 21;2:147. doi: 10.1038/s43856-022-00215-3 (PMC9678896; doi:10.1038/s43856-022-00215-3)
Supplement: Supplementary file 2 — Supplementary Information [file 43856_2022_215_MOESM2_ESM.pdf]

Dark-field Chest X-ray Imaging for the Assessment of COVID-19-Pneumonia

Supplementary Information

Authors: Manuela Frank[1,2,3*†], Florian T. Gassert[3†], Theresa Urban[1,2,3], Konstantin Willer[1,2,3], Wolfgang Noichl[1,2], Rafael Schick[1,2,3], Manuel Schultheiss[1,2,3], Manuel Viermetz[1,2], Bernhard Gleich[2], Fabio De Marco[1,2], Julia Herzen[1,2], Thomas Koehler[4,5], Klaus Jürgen Engel[4], Bernhard Renger[3], Felix G. Gassert[3], Andreas Sauter[3], Alexander A. Fingerle[3], Bernhard Haller[6], Marcus R. Makowski[3], Daniela Pfeiffer[3,5], Franz Pfeiffer[1,2,3,5]

Affiliations:
[1]Chair of Biomedical Physics, Department of Physics, School of Natural Sciences, Technical University of Munich, 85748 Garching, Germany.
[2]Munich Institute of Biomedical Engineering, Technical University of Munich, 85748 Garching, Germany.
[3]Department of Diagnostic and Interventional Radiology, School of Medicine & Klinikum rechts der Isar, Technical University of Munich, 81675 München, Germany.
[4]Philips Research, Hamburg, Germany.
[5]Institute for Advanced Study, Technical University of Munich, 85748 Garching, Germany.
[6]Institute of AI and Informatics in Medicine, School of Medicine & Klinikum rechts der Isar, Technical University of Munich, 81675 München, Germany.

*Correspondence to: manuela.frank@tum.de
†These authors contributed equally to this work.

Supplementary Figure 1 | Comparison of Dark-field chest X-ray and CT information for a selected patient.

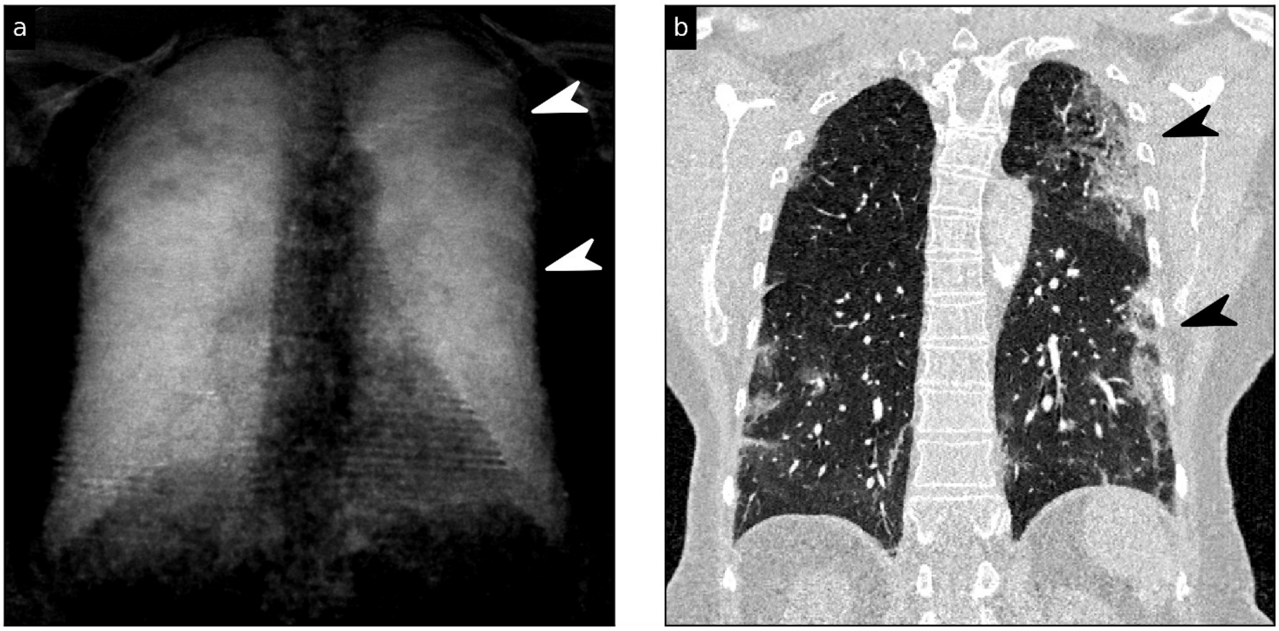

***a**, Dark-field chest X-ray radiograph and **b**, exemplary coronal CT slice of a 67-year-old female patient infected with COVID-19. The dark-field chest X-ray shows an overall signal reduction and an inhomogeneous structure. The patchy appearance in the periphery in the dark-field image corresponds well to the ground glass opacities and consolidated areas in the CT scan (arrowheads).*

Supplementary Table 1 | Sensitivity, specificity, and accuracy for each reader individually and overall.

	Reader	Sensitivity	Specificity	Accuracy
Attenuation	1	0.50	0.84	0.63
	2	0.33	0.98	0.59
	3	0.38	1.00	0.63
	4	0.48	0.98	0.68
	Overall	0.43	0.93	0.63
Dark-field	1	0.92	0.95	0.93
	2	0.95	0.90	0.93
	3	0.92	0.88	0.90
	4	0.65	0.78	0.70
	Overall	0.86	0.84	0.85
Attenuation & Dark-field	1	0.93	0.97	0.95
	2	0.90	0.95	0.92
	3	0.88	0.95	0.91
	4	0.80	0.86	0.82
	Overall	0.88	0.89	0.89

Supplementary Table 2 | Inter-rater reliability expressed with Cohen's quadratic weighted kappa.

	Attenuation	Dark-field	Attenuation & Dark-field
Reader 1 & Reader 2	0.27	0.56	0.48
Reader 1 & Reader 3	0.38	0.67	0.63
Reader 1 & Reader 4	0.36	0.57	0.59
Reader 2 & Reader 3	0.16	0.62	0.74
Reader 2 & Reader 4	0.22	0.65	0.66
Reader 3 & Reader 4	0.42	0.66	0.74
